# Affinity Effects on the Release of Non-Conventional Antifibrotics from Polymer Depots

**DOI:** 10.3390/pharmaceutics12030275

**Published:** 2020-03-17

**Authors:** Nathan A. Rohner, Dung Nguyen, Horst A. von Recum

**Affiliations:** 1 Department of Biomedical Engineering, Case Western Reserve University, 10900 Euclid Ave., Cleveland, OH 44106, USA; nathan.rohner@case.edu; 2 Department of Macromolecular Science and Engineering, Case Western Reserve University, 2100 Adelbert Road, Cleveland, OH 44106, USA; dvn9@case.edu

**Keywords:** controlled release, chronic disease, pharmaceuticals, small molecules, localized therapy

## Abstract

For many chronic fibrotic conditions, there is a need for local, sustained antifibrotic drug delivery. A recent trend in the pharmaceutical industry is the repurposing of approved drugs. This paper investigates drugs that are classically used for anthelmintic activity (pyrvinium pamoate (PYR)), inhibition of adrenal steroidgenesis (metyrapone (MTP)), bactericidal effect (rifampicin (RIF), and treating iron/aluminum toxicity (deferoxamine mesylate (DFOA)), but are also under investigation for their potential positive effect in wound healing. In this role, they have not previously been tested in a localized delivery system suitable for obtaining the release for the weeks-to-months timecourse needed for wound resolution. Herein, two cyclodextrin-based polymer systems, disks and microparticles, are demonstrated to provide the long-term release of all four tested non-conventional wound-healing drugs for up to 30 days. Higher drug affinity binding, as determined from PyRx binding simulations and surface plasmon resonance in vitro, corresponded with extended release amounts, while drug molecular weight and solubility correlated with the improved drug loading efficiency of cyclodextrin polymers. These results, combined, demonstrate that leveraging affinity interactions, in combination with drug choice, can extend the sustained release of drugs with an alternative, complimentary action to resolve wound-healing and reduce fibrotic processes.

## 1. Introduction

In many cases, from rheumatic diseases to liver fibrosis, unresolved wound healing can contribute to fibrosis [1,2]. Fibrosis may affect a range of internal organs with little indication until later stages. This can necessitate surgery, lead to hospital readmission and introduce other costly complications. Better understanding of the pathways involved in wound resolution is increasing the opportunities for alternative uses of United States Food and Drug Administration approved pharmaceuticals to improve wound healing and reduce or even prevent fibrotic progression. However, current small molecules are limited in their delivery route and bioavailability due to quick clearance from circulation and short metabolic half-lives [3,4]. Moreover, patient compliance and dosing regimens to maintain effects may be impractical for the wound resolution timeline, which can span months [5,6]. In this study, we examine polymer depots and drug combinations which can extend therapeutic effect to timelines matching wound resolution to improve anti-fibrotic outcomes.

Previous work has demonstrated the use of modified cyclodextrins and cyclodextrin polymers to deliver poorly soluble drugs [7,8,9]. However, using cyclodextrins (CD) in a different line of work, we sought to provide a sustained delivery of small molecules for longer than their short, diffusion-based half-lives to better match the timeline of fibrosis and wound healing. Thus, we developed insoluble polymer depots with a high concentration of CD units to support repeated inclusion complex formation and sustain release based upon the drug–CD affinity interactions, which have been previously leveraged in antibiotic and anti-inflammatory applications [10,11,12,13]. While RIF has shown extended delivery via affinity-based methods [14,15], the release profiles of DFOA, MTP, and PYR using affinity-based polymers have not been studied (Table 1). Additionally, depot formulation as either disks (large, continuous polymers, ~6 mm in diameter with greater void space) versus microparticles (smaller, ~15 microns discrete and compact polymer spheres) is explored in this study (Supplementary Figure S1).

## 2. Materials and Methods

### 2.1. Materials

β-cyclodextrin (β-CD) prepolymer, lightly crosslinked with epichlorohydrin, and heptakis (6-deoxy-6-amino) β-cyclodextrin heptahydrochloride were purchased from CycloLab (Budapest, Hungary). Ethylene glycol diglycidyl ether was purchased from Polysciences, Inc. (Warrington, PA, USA). Dextran (15–25k molecular weight) and hexamethylene diisocyanate were purchased from Sigma-Aldrich (St. Louis, MO, USA). Small molecules used were pyrvinium pamoate (US Pharmacopeia, Rockville, MD, USA), metyrapone (Enzo Life Sciences, Farmingdale, NY, USA), rifampicin (Research Products International, Mt. Prospect, IL, USA), and deferoxamine (Millipore Sigma, Burlington, MA, USA). All other reagents, solvents, and chemicals were purchased from Thermo Fisher Scientific (Waltham, MA, USA).

### 2.2. Molecular Docking Simulations

Molecular structure data files for deferoxamine (CID: 2973), rifampicin (CID: 5997), metyrapone (CID: 4174), pyrvinium (CID: 5281035) and β-CD (CID: 444041) were downloaded from the PubChem database. Structures were converted to PDBQT format. β-CD was used as a host for small molecule docking in PyRx (Molecular Graphics Laboratory, The Scripps Research Institute, La Jolla, CA, USA). The strength of the interaction was predicted using the Autodock Vina algorithm [26,27,28,29].

### 2.3. Surface Plasmon Resonance

The binding strength between a β-CD monomer with either DFOA, RIF, MTP, or PYR was measured experimentally through surface plasmon resonance (SPR) using the Biacore X100 system (GE Healthcare Bio-Sciences, Pittsburgh, PA, USA). Surface optimization for small molecule drugs was performed via previously established conjugation of cyclodextrins [12,30]. The surface of a sensor chip CM-5 was conjugated with (1-ethyl-3-(3-dimethylamino) propyl carbodiimide, hydrochloride (0.4 M) and N-hydroxysuccinimide (0.1 M) followed by 10 mM 6-amino-6-deoxy β-cyclodextrin suspended in HBS-N buffer (a HEPES balanced salt solution with pH 7.4). The surface was capped with ethanolamine. A multi-cycle kinetic experiment was performed separately with the following analyte solutions and running buffers: DFOA in diH2O, RIF in diH2O, MTP in diH2O and PYR in diH2O with 5% dimethylsulfoxide. The surface was regenerated with 50 mM sodium hydroxide between samples to remove any remaining analyte. The response data were fit to both steady state affinity and a 1:1 kinetics binding model using Biacore evaluation software. The goodness of fit was determined by U-value <25, as specified in the manufacturer’s protocols.

### 2.4. Disk Synthesis

Epichlorohydrin-crosslinked β-CD prepolymer (or dextran for non-inclusion complex forming comparison) was dried under vacuum and then dissolved in dimethylsulfoxide at a 25% ***w/v*** solution under heated stirring. 1,6-hexamethylene diisocyanate was added and the solution vortexed for 2 min. The solution was spread upon a Teflon dish and protected from air to crosslink until solidified. A circular biopsy punch was used to make disks which were washed in sequence with excess dimethylsulfoxide for one day, 50:50 dimethylsulfoxide and deionized water the next day, then deionized water alone for 3 days before drying [31].

### 2.5. Microparticle Synthesis

As previously performed [32], epichlorohydrin-crosslinked β-CD prepolymer (or dextran for non-inclusion complex-forming comparison) was solubilized in 0.2 M potassium hydroxide (25% *w/v*) and heated to 60 °C for 10 min. Light mineral oil in a beaker was warmed with a Tween85/Span85 solution (24%/76%) and mixed on a stir plate. Next, ethylene glycol diglycidyl ether was added drop-wise and the solution was vortexed before pouring into the oil/Span/Tween85 mixture, increasing the temperature to 70 °C. The polymerized cyclodextrin microparticles were formed after 3 h. The microparticles were then centrifuged from the oil mixture, washed with excess hexanes twice and deionized water twice. The microparticles were resuspended, frozen, and lyophilized to complete dryness before further use.

### 2.6. Drug Loading and Release

Either DFOA, MTP, PYR, or RIF was loaded in a 1:4 (drug:polymer ratio) solution with a final loading concentration of 5 mg/mL drug for 72 h on a rotary shaker. Loading solutions were removed and particles were washed and then mixed with release buffer (1× Dulbecco’s phosphate buffered saline with 0.1% Tween80) to provide physiological buffering and a hydrophobic sink [33,34]) and incubated at 37 °C on a rotary shaker. At a pre-determined timepoint of 1 h and daily thereafter, the particles were centrifuged and a release buffer was exchanged to monitor drug release and maintain physiological sink conditions. Drug concentrations were determined with a Synergy H1 Hybrid Multi-Mode Microplate Reader (BioTek Instruments, Inc., Winooski, VT, USA) by measuring the absorbance of the individual release aliquots at 240 nm for DFOA, 260 nm for MTP, 475 nm for RIF, and 510 nm for PYR in a quartz microplate (Hellma, Plainview, NY, USA) and comparing to the respective standard curve in release buffer.

### 2.7. Statistics

The compiled data are represented as the mean with standard deviation or standard error of the mean where specified. Statistics were calculated using GraphPad Prism 8 software (GraphPad Software, La Jolla, CA, USA). Statistical significance was defined as *p* < 0.05 following statistical tests, with additional specifications listed in the respective figure captions. Pearson “r” values were calculated for correlation coefficients.

## 3. Results

### 3.1. In Silico Affinity Binding Predictions

**Figure 1:**
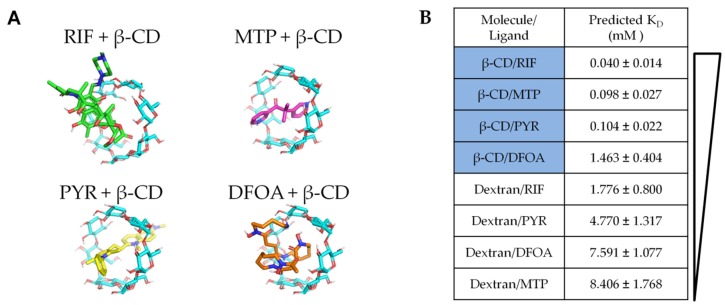
(**A**) Molecular structures of rifampicin (RIF), metyrapone (MTP), pyrvinium (PYR), and deferoxamine (DFOA) represented in 3D with JMOL software showing relative size and potential binding conformations for the β-CD inclusion complex formation. (**B**) PyRx simulations were performed to predict the binding affinity of drugs with β-CD versus dextran as a chemically similar, but non-inclusion-forming, control, as dextran lacks the binding cavity of β-CD.

To first predict the relative binding affinities of the chosen drugs for the β-CD versus dextran (chemically similar control without inclusion complex), a molecular docking program was utilized (PyRx with Autodock Vina software [28,35]). The β-CD monomer, dextran, and drug molecule structure files were downloaded from online databases as specified in the methods section (Figure 1A). DFOA, RIF, MTP, and PYR were individually simulated to determine minimal binding energy configurations with either β-CD or dextran as the target host macromolecule. The results were converted to dissociation constants for each simulated pair.

The lowest K_D_ (highest binding affinity) was predicted for RIF and β-CD (0.04 mM), while the K_D_ for MTP and PYR β-CD complexes were more than twice as large at 0.098 and 0.104 mM, respectively (Figure 1B). DFOA resulted in the highest K_D_ out of all the β-CD simulations at 1.463 mM. When the drugs were simulated with dextran, the K_D_ values were higher. The combination of RIF and dextran demonstrated a K_D_ of 1.776 mM, followed by PYR-dextran (4.770 mM), DFOA-dextran (7.591 mM), and MTP-dextran (8.406 mM). The differences between dextran and β-CD affinities were expected from previous studies as dextran is chemically similar but with a linear/branched structure rather than the ring shape of β-CD [30,36]. Herein, these results predict that RIF will have the greatest affinity for polymers synthesized from β-CD, while DFOA will have the least affinity.

### 3.2. In Vitro Affinity Measurements with Surface Plasmon Resonance

**Figure 2:**
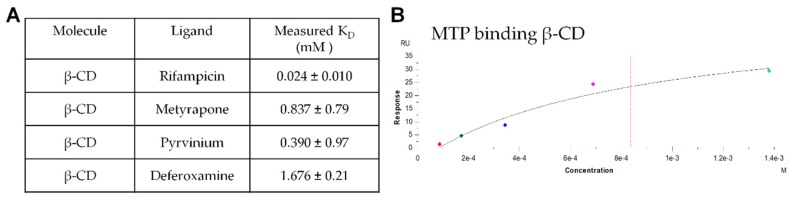
(**A**) The binding affinity of RIF, MTP, PYR and DFOA with surface-conjugated β-CD was experimentally determined by surface plasmon resonance. (**B**) Example binding response evaluation for metyrapone flowing over and binding to the CM5 chip surface coated with β-CD.

Next, the binding affinity of the drug molecules to β-CD was analyzed in vitro by SPR to verify the simulation results. The individual drug analytes flowing over the chip surface conjugated with 6-amino-6-deoxy-β-CD were compared to the dextran reference channel, and the resulting binding curves were analyzed with the built-in software. Results in Figure 2A demonstrated that RIF had the greatest binding affinity (0.024 mM, lowest K_D_), similar to the in silico results from PyRx. While MTP and PYR had slightly higher K_D_ values that were within error of the simulation and DFOA maintained the highest K_D_. The example binding curve shows the curve fit for MTP binding to β-CD with the other binding curves generated were of similar quality (Figure 2B).

### 3.3. Drug Loading Efficiencies

**Figure 3:**
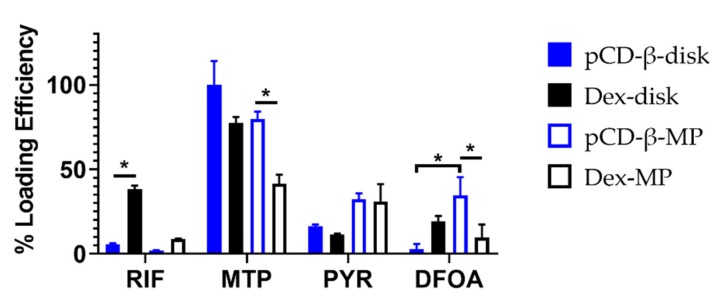
Loading efficiency for drugs into disk and microparticle polymers synthesized of either β-CD or dextran. * indicates *p* < 0.05 by two-way ANOVA with Tukey’s test post-hoc; n = 3 each.

The polymers were loaded with drugs, individually, over 72 h before being washed and then transferred to a new tube with a release buffer. Drugs remaining in the polymer were leeched over time to quantify the loading efficiencies of the polymer–drug combinations. In Figure 3, trends showed that polymerized β-CD disks (pCD-β-disk) had improved loading efficiency for MTP and PYR over the lower affinity dextran disks (Dex-disk). However, RIF and DFOA both loaded with greater efficiency into the Dex-disk than the pCD-β-disk. Comparing the microparticle polymers, significantly more MTP and DFOA were loaded into pCD-β-MP versus Dex-MP (Figure 3). PYR loaded similarly between the pCD-β-MP and Dex-MP, while RIF showed a trend towards higher Dex-MP loading.

### 3.4. Daily Drug Release Profiles

**Figure 4:**
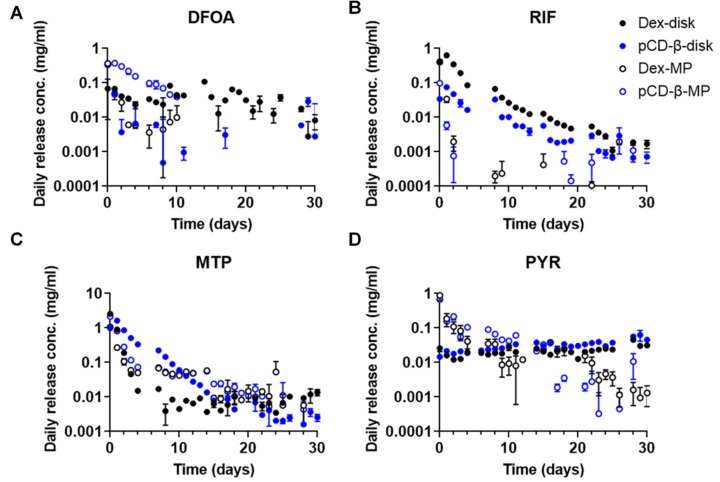
Daily release concentrations for (**A**) DFOA, (**B**) RIF, (**C**) MTP, and (**D**) PYR from 20 mg of either pCD-β-disks, Dex-disks, pCD-β-MP, or Dex-MP. n = 3 each plotted with SEM. Note the *y*-axis is log-scaled to demonstrate sustained differences, but a linear-scaled version is available as Supplementary Figure S2.

Measuring daily release aliquots with continuous replacement allowed us to determine the rate and amount of drug released from each type of polymer depot in a physiological scenario. Drug aliquot absorbances were compared to individual standard curves created in release buffer to calculate concentrations. The greatest differences in release concentration were observed within the first few days, consistent with the loading trends observed in the previous section. For DFOA, disks released over a longer time than the microparticles (Figure 4A). β-CD microparticles exhibited a greater daily concentration of DFOA released than the dextran microparticles, yet the trend was reversed for the disks. More RIF was delivered by disks than microparticles, with dextran polymers delivering greater concentrations than β-CD (Figure 4B). RIF was continuously delivered from all polymer forms for the 30 days of the study. A more gradual, sustained release of MTP from β-CD versus dextran disks was noted (Figure 4C). While MTP release rates were similar between β-CD and dextran microparticles, the overall amounts were greater for the affinity-based β-CD depot (Figure 4C). PYR release from disks was relatively constant over the 30-day study, while release from microparticles decreased over time, similar to the other observed trends (Figure 4D).

### 3.5. Cumulative Drug Release

**Figure 5:**
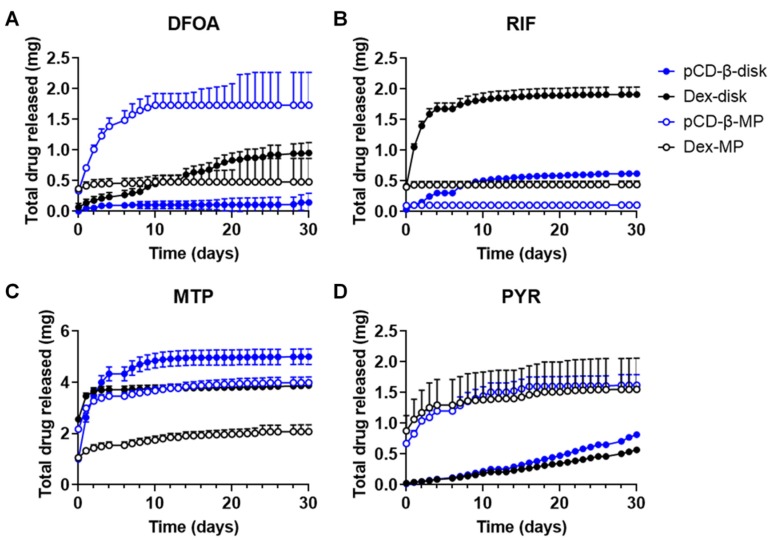
Cumulative drug release calculated for (**A**) DFOA, (**B**) RIF, (**C**) MTP, and (**D**) PYR from pCD-β-disks, Dex-disks, pCD-β-MP, and Dex-MP. n = 3 each plotted with SEM. A total of 5 mg of drug was initially loaded into 20 mg of polymer for each sample.

In order to analyze the total doses of drugs delivered from the polymers, the cumulative amount was calculated from the daily release data. These graphs inform how much drug is released overall, as well as the relative rate of the burst versus sustained release for each polymer–drug combination studied. DFOA exhibited the greatest release from β-CD microparticles, plateauing over a period of 10 days, while the dextran microparticles exhibited a short burst of DFOA release (Figure 5A). However, dextran disks sustained a longer and larger amount of DFOA release than β-CD polymer disks. The total amount of RIF released was highest from the dextran disks, but β-CD disks exhibited a more sustained release, that increased over the 30-day period (Figure 5B). Given the conditions and loading concentrations of this study, both β-CD and dextran microparticle RIF release reached a plateau early in the study with dextran microparticles releasing a greater total amount. MTP release from β-CD disks was the highest of all drug–polymer combinations, exhibiting a quicker initial release than the β-CD microparticles, but both sustained release for up to 30 days (Figure 5C). The total cumulative release of MTP was greater for β-CD than respective dextran polymers (1.3x for disks and 1.9x for microparticles). For PYR, the cumulative release curves were similar among the β-CD and dextran polymers, with microparticles releasing more in the early timepoints and disks exhibiting a more sustained release, consistent with previous affinity-based results for other hydrophobic drug molecules [37,38,39] (Figure 5D).

### 3.6. Drug-Polymer Characteristics Juxtaposed with Release Data

The combined effects of both drug and polymer properties can influence delivery from polymer depots. In this study, the inclusion complex of β-CD is hypothesized to influence drug release profiles. MTP was the smallest drug of the chosen molecules and also exhibited the greatest difference in relative β-CD versus dextran affinity (indicating a higher proclivity for association with β-CD), which corresponded with the highest loading efficiency and maximum drug released (Table 2). While no drug exhibited a β-CD/dextran affinity over 1, the highest was DFOA at 0.193. However, DFOA release from both β-CD and dextran polymer disks were consistently non-zero for 30 days. While PYR and RIF had similar β-CD/dextran affinity ratios, PYR was the most hydrophobic molecule and exhibited the longest sustained release profile, with consistent release for the 30-day study from all polymer forms. RIF release quickly reached a plateau from the microparticles, yet the β-CD disk continued to sustain RIF release until day 30. With the β-cyclodextrin disks utilized in this study, the molecular weight and hydrophobicity of the drugs were found to correlate more closely with loading efficiency (Pearson correlation coefficients of r = −0.761 and r = −0.555, respectively [40]) than the affinity measurements (correlation coefficient of r = −0.390). Binding affinity correlated more with drug release rate from cyclodextrin-based polymers (r = −0.283) than molecular weight with release rate (r = −0.145), while solubility maintained a larger effect on the release half-life (r = −0.490). Similarly, with the smaller β-cyclodextrin microparticles, loading efficiency correlated with molecular weight (r = −0.918) and solubility (r = −0.375) of the drug rather than affinity binding (r = −0.023). Together, these results indicate a predicted improvement in drug-loading into cyclodextrin-based polymers for smaller and more hydrophobic drugs, while the release rate is dependent on affinity and drug solubility. Overall, many of the polymer–drug combinations were able to extend drug release for 30 days, indicating their potential use in localized wound-healing depots (Table 2). Still, the use of single molecule affinity interactions are limited in their predictive capacity for the performance of a polymer network. Multiple binding interactions, such as in molecularly imprinted polymers, may further sustain drug release or improve drug-loading efficiency and specificity [41,42,43].

## 4. Discussion

The small molecule drugs selected for the present study all have alternative potentials to improve wound-healing and reduce or prevent fibrosis (Table 1). Of particular interest is the relative effect of affinity binding on the sustained release of drugs for chronic applications. We herein used both in silico and in vitro measurements to predict and confirm the relative affinity of the selected drugs for β-CD or the base polymer without inclusion complex formation, dextran (Figure 1 and Figure 2). Previous studies have demonstrated using cyclodextrin monomers polymerized into larger macrostructures such as coatings, disks, and particles in order to delay drug release by achieving a high concentration of affinity-binding sites [27,30,31,37,44,45]. The release results herein show differences in the loading efficiencies (Figure 3), release rates (Figure 4), and cumulative amounts of drug release (Figure 5) which may inform the future use and design of local drug delivery depots. As the timeline of wound-healing in many applications is on the order of months, the ability to sustain drug delivery for a similar time period (Table 2) can improve outcomes and potentially reduce detrimental fibrosis and scarring [36]. In addition to selecting polymer/drug combinations which are experimentally known to provide greater release, results indicate that improving the affinity or solubility of drugs with the cyclodextrin-based polymers could improve drug-loading efficiency and total release amounts. Previous studies have shown the incorporation of high-affinity adamantane modifications to extend doxorubicin delivery from cyclodextrin polymer depots for up to 87 days [46]. Future work could incorporate multiple different binding sites to improve the delivery of drug cocktails for various wound-healing and anti-fibrotic applications (e.g., from the current results, a co-polymer with both β-CD and dextran domains may result in better co-delivery of RIF and MTP than either β-CD or dextran alone). Incorporating native structures, such as albumin or heparin, in future devices could allow for co-delivery of growth factors, cytokines, or other signaling molecules for improved efficacy [27,47]. Furthermore, the diisocyanate-crosslinked CD polymer depots are known to be refillable in vivo [15,48], even through biofilms and in the presence of serum molecules [10,26], yet the effects of fibrotic processes such as collagen deposition and immune system activity on drug refilling into cyclodextrin polymer depots have yet to be determined.

The use of virtual screening in drug discovery cuts the time and costs of finding new potential therapies [49]. Leveraging these software and binding equations, the affinity of drug molecules for candidate polymers can be pre-assessed to speed translation for affinity-based delivery systems (an advantage over standard degradable polymer matrices or diffusion-reliant systems). However, as experiments that test sustained drug delivery can last for months in chronic disease or wound-healing applications, a quick simulation can be a powerful design tool. In this study, we noted that, despite relatively high single-molecule-binding predictions with a β-CD monomer, the binding affinity of drugs did not correlate well with drug loading and were only moderately predictive of a slower release rate (Figure 1, Figure 3, and Figure 5). This result is partially expected, as molecular docking and energy minimization for single-molecule interactions cannot hope to predict the complex multitude of interactions that occur with polymer drug delivery. To overcome this challenge, better methods are in development for more powerful quantitative structure–activity relationship (QSAR) approaches to predict drug/material interactions, for improving delivery rates to match disease timelines [35]. The drug properties which correlate with improved release rates and loading efficiencies in this work would then become strong descriptors (physical or chemical characteristics) in a dataset for selecting better antifibrotic drug candidates; for example, narrowing the study to drugs with similar descriptors as MTP with the assistance of database searching and machine learning [35].

Moreover, the identification of key descriptors and the use of predictive software could lead to new chemistries for extending drug delivery. Beyond using the traditional adamantane-CD bond for improved drug delivery [46,50], recent work has shown that molecular tethering of multiple drug molecules can improve interactions with CD-based polymers [51]. Specifically, the delivery window for rapamycin, traditionally a quickly-diffusing and relatively insoluble drug, was tripled to 65 days of release in a “dimeric” formulation versus the standard drug molecules, while maintaining antifibrotic activity [51]. Thus, by modifying the drug itself for more favorable interaction with CD polymers, future work can overcome delivery limitations and improve therapeutic outcomes. 

## 5. Conclusions

In this study, polymer microparticles and disks were synthesized from either inclusion-complex-forming β-CD or linear/branched dextran. Affinity interactions were predicted with PyRx and measured by SPR between a selection of non-conventional wound-healing drugs and the β-CD or dextran pre-polymer subunits. Results demonstrated that the small molecule drug MTP, which exhibited the highest binding affinity ratio, also maintained the highest release profile. The release of PYR from polymer disks was the most linear over time, in some part due to hydrophobic interactions. At least one polymer–drug combination for each selected drug was able to continuously release the drug for 30 days, indicating the potential of affinity-based polymers to extend delivery over the time course of wound resolution and reduce fibrotic processes.

Our results provide insights into the usefulness of other drug descriptors in the selection of drug/material combinations for affinity-based drug delivery, apart from single-molecule-binding affinity. As polymer depots contain a multitude of subunits, the interaction of many polymer domains with internal drug molecules is complex, yet herein we observed smaller molecular weight and drug hydrophobicity to coincide with improved drug-loading in CD polymers. Additionally, affinity interactions and drug solubility were moderately correlated with drug release rate. Taking into consideration the many possible descriptors to be evaluated for future studies, our results indicate the value of developing future tools to better predict polymer/drug interactions for delivery rates to match disease timelines and improve therapeutic outcomes.

## Figures and Tables

**Table 1:** The conventional use and route of small molecule drugs which are also under investigation for potential positive effects in wound-healing resolution and reducing fibrosis.

Drug	Conventional Use	Conventional Route	Wound Resolution Indications
Deferoxamine (DFOA)	Treating iron/aluminum toxicity	SQ, IM, IV	Decreases oxidative stress and necrosis [16] Enhances neovascularization [17] Reduces interstitial renal fibrosis [18]
Metyrapone (MTP)	Inhibition of adrenal steroidgenesis	PO	Enhances epithelialization [19,20]
Rifampicin (RIF)	Antibiotic activity	PO, IV	Ulcer resolution [21] Immunomodulatory [22,23]
Pyrvinium pamoate (PYR)	Anthelmintic activity	PO	Wnt inhibition [24] Inhibits fibroblast survival [25]

**Table 2:** Properties of DFOA, MTP, PYR, and RIF and results of polymer drug release studies. The fold change in affinity due to inclusion complex was calculated from PyRx results (lower value indicates greater affinity binding). Maximum total drug released, and days of continuous, above assay background release are listed above the corresponding polymer type per each drug.

Drug	Mol. Wt.(g/mol)	Solubility (PBS, 25 °C, mg/mL)	β-CD/Dex Affinity Ratio	Max Total Release (mg)	Days of Release
Deferoxamine	560.68	5.0	0.193	1.7 ± 0.92	30
				pCD-β-MP	Disks
Metyrapone	226.27	0.43	0.012	5.0 ± 0.53	30
				pCD-β-disk	All polymers
Pyrvinium	382.52	0.00029	0.022	1.6 ± 0.30	30
				pCD-β-MP	All polymers
Rifampicin	822.94	2.5	0.023	1.9 ± 0.21	30
				Dex-disk	Disks

## Supplementary Materials

The following are available online at https://www.mdpi.com/1999-4923/12/3/275/s1.
