# Supplementary material for: Affinity Effects on the Release of Non-Conventional Antifibrotics from Polymer Depots
Source: Pharmaceutics. 2020 Mar 17;12(3):275. doi: 10.3390/pharmaceutics12030275 (PMC7151100; doi:10.3390/pharmaceutics12030275)
Supplement: Supplementary file 1 [file pharmaceutics-12-00275-s001.pdf]

pharmaceutics

MDPI

Article

Affinity Effects on the Release of Non-Conventional Antifibrotics from Polymer Depots

Nathan A. Rohner [1], Dung Nguyen [2] and Horst A. von Recum [1,*]

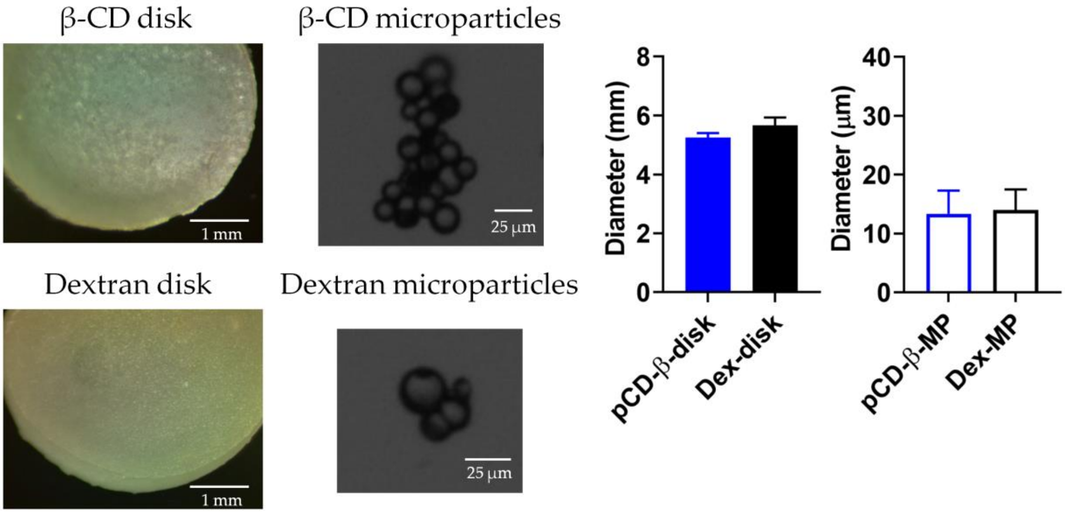

Figure S1. Stereomicroscope images of synthesized polymer disks and microscope images of polymer microparticles showing similarity in structures. Quantification is represented as mean with standard deviation of n=8 measurements for disks and n=40 for microparticles.

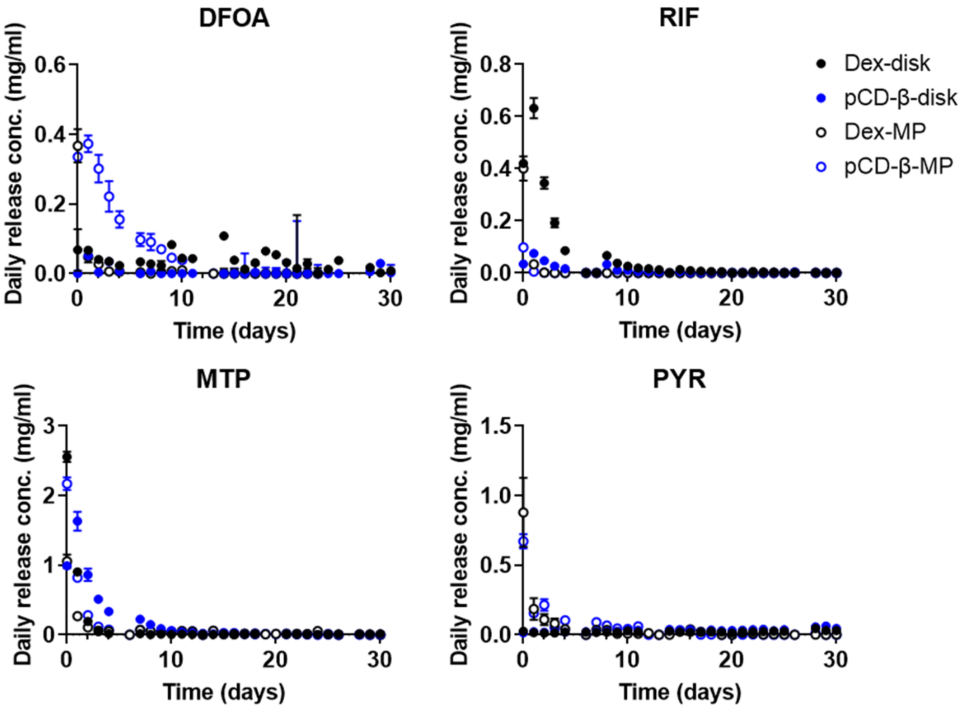

Figure S2. Daily drug release concentrations from Figure 4 are plotted with linear y-axis scaling as an alternative presentation. While this graph demonstrates more clear differences in the early release phase, the data at later timepoints (low concentration, sustained release) are better depicted in the log-scaled Figure 4.
